# Acromioclavicular Fixation Before Coracoclavicular Tunnel Placement and Acromioclavicular Construct Design Improved Reduction and Stability in a Whole-Shoulder Girdle Model: A Pilot Study

**DOI:** 10.1177/03635465251349143

**Published:** 2025-06-26

**Authors:** Nicolas Holzer, Pascal Boileau, Toby Baring, Jean-Yves Beaulieu, Noria Foukia, Michel Lauria, Stéphane Armand, Florent Moissenet

**Affiliations:** † Department of Orthopaedics and Trauma Surgery, Geneva University Hospitals, Geneva, Switzerland; ‡ Faculty of Medicine, University of Geneva, Geneva, Switzerland; § Institut de Chirurgie Réparatrice Locomoteur & Sport–Nice, Nice, France; ‖ Department of Orthopaedics, Homerton University Hospital, London, United Kingdom; ¶ Geneva School of Engineering, Architecture and Landscape–HEPIA, Geneva, Switzerland; Investigation performed at Geneva University Hospitals, Geneva, Switzerland

**Keywords:** acromioclavicular lesion, surgical step sequence, bracing construct design, joint kinematics, cadaveric specimen, robotic manipulator, whole shoulder

## Abstract

**Background:**

Reconstruction of the acromioclavicular (AC) ligament after an acute AC dislocation as the first surgical step before coracoclavicular (CC) tunnel placement has been proposed to reduce the risk of postoperative loss of reduction. Additional reconstruction of AC ligamentous complex lesions with different types of bracing constructs has also been described to improve outcomes. Still, the effect of the sequence of surgical steps and the AC bracing construct design on the AC kinematics in a whole-shoulder girdle model has not been reported.

**Hypothesis:**

The primary hypothesis was that postoperative AC joint reduction would improve when the AC joint was reconstructed before CC tunnel placement. The secondary hypothesis was that different AC bracing construct designs affect joint kinematics during physiological motion in a whole-shoulder girdle model.

**Study Design:**

Controlled laboratory study.

**Methods:**

Five cadaveric specimens (10 shoulders) were prepared for whole-shoulder mobilization with a robotic manipulator. Joint kinematics was acquired during physiological motions using an optical motion capture system. Recorded parameters were (1) the joint reduction in a resting position, expressed as joint displacements and rotations compared with an intact AC joint, and (2) the joint stability during all tested motions, expressed as joint displacements and rotations. The tested joint conditions were intact AC joint, induced Rockwood type 5 lesion, isolated CC reconstruction, and 4 AC joint bracing construct designs. AC reconstruction was performed before (AC-first technique) and after (CC-first technique) CC tunnel placement in 5 shoulders each.

**Results:**

The AC-first surgical step improved the AC joint reduction in anterior-posterior tilt compared with CC-first (median difference, −9.4°; *P* < .001). The AC-first surgical step also demonstrated an increased superior-inferior joint reduction with hyperreduction (median difference, 1.6 mm; *P* = .041) compared with CC-first. Dispersion of joint reduction values was reduced with the AC-first step and particularly for anterior-posterior tilt (IQR difference, −4.8°) and lateral-medial displacement (IQR difference, −3.4 mm). The double vertical bracing construct design increased the AC joint stability compared with other constructs and reached a statistical significance in all rotational displacement (*P* < .001 to *P* = .041) as well as in lateral-medial displacement (*P* = .001 to *P* = .015).

**Conclusion:**

The AC-first surgical step sequence improved AC joint alignment in the scapular sagittal plane and increased joint hyperreduction. The double vertical bracing construct design achieved the highest joint stability over other tested designs during passive motion.

**Clinical Relevance:**

The restoration of the preinjury joint alignment and the optimization of the joint stability may improve outcomes and reduce the risk of construct de-tensioning during the rehabilitation phase.

Injuries of the acromioclavicular (AC) joint are common, with a reported incidence of 2.0 per 100,000 in the general population^26^ and 9.2 per 1000 in young athletes.^29^ A therapeutic management is currently recommended based on the assessment of kinematic alterations in shoulder girdle movements.^4^ Overriding of the clavicle and scapular dyskinesia resistant to nonoperative treatments has been proposed as the main criteria for surgery in chronic cases. The goals of surgical reconstruction in our study were defined as (1) restoration of the anatomic joint alignment and (2) an internal fixation providing a temporary joint stabilization to allow capsuloligamentous healing. A consensus for treatment was released in 2019 by the European Shoulder Associates–European Society of Sports Traumatology, Knee Surgery and Arthroscopy.^33^ High-grade acute lesions were recommended to be managed with an arthroscopically assisted anatomic reconstruction. A previous clinical multicentric study conducted by the French Society of Arthroscopy recommended stabilization of high-grade acute lesions by combining AC and coracoclavicular (CC) constructs.^2^

Nevertheless, surgical management remains a controversial issue. Strategies may vary in terms of reconstruction type (CC ± AC reconstruction), surgical step sequence,^37^ and bracing construct design.^3^ The sequence of CC and AC surgical steps has been shown to affect both clinical and radiological outcomes.^37^ The initial AC joint reduction was hypothesized to improve CC tunnel alignment, potentially explaining the observed optimization of radiological outcomes expressed as the CC distance ratio and tunnel widening. This hypothesis has not yet been confirmed by a controlled laboratory study. Numerous bracing construct designs for AC joint reconstruction have been reported. The biomechanics of these constructs has been predominantly studied on uniaxial bone loading models with neutralization of scapular motions.^42^ With the use of such models, the addition of AC bracing constructs to CC suspension devices has been shown to increase the anterior-posterior joint stability of the isolated clavicle in relationship to a fixed scapula.^#^ The kinematics of a set of AC bracing construct designs was assessed by Dyrna et al.^12^ They reported that the native anterior-posterior joint stability could be restored by the addition of an AC capsule augmentation, while partial rotational instability remained. However, their results did not reveal differences between bracing construct designs.

While these studies highlighted the effect of the surgical step sequence and bracing construct design on isolated AC joints, their potential effects on the complete-shoulder girdle kinematics remain uninvestigated. However, complete-shoulder girdle models have been proposed as standard to study AC joint lesions.^27,28,30-32,43^ The sequential AC ligament sectioning study by Oki et al^27,28,30-32,43^ concluded that AC and CC injuries could lead to scapular dyskinesia. The kinematic modifications were accordingly proposed to be the underlying cause of AC joint injury–associated symptomatology. The authors also highlighted the negative effects of the unstable joint leading to overriding of the clavicle superior to the acromion.^27^ The proposed whole-shoulder girdle model has been established in further studies; Pastor et al^30^ studied the effect of the deltotrapezoid fascia lesion on joint kinematics. Walley et al^43^ reported significant modifications of the glenohumeral joint center displacements starting at a Rockwood type 3 lesion. To the best of our knowledge, only Peeters et al^32^ assessed AC joint reconstructions with such a model. The authors compared AC, CC, or combined reconstructions, concluding that none of these strategies could restore shoulders to the preinjury state.

As complications and clinical failure rates remain a matter of concern,^8,10,21,22,34,38,40,44^ further research to restore the native kinematics of the AC joint after significant injury has been advocated.^4^ Based on a whole-shoulder girdle model, this pilot study aimed to conduct further comparative analyses on the role of the surgical step sequence and bracing construct design in AC joint reconstruction, and to assess their effect on joint alignment and stability. Our primary hypothesis was that reconstruction of the AC joint before CC tunnel placement would improve reduction. Our secondary hypothesis was that different types of AC bracing construct designs would affect joint kinematics during physiological motion.

## Methods

### Specimen Preparation

Five fresh-frozen, unembalmed adult whole cadavers (77.4 ± 9.99 years) were obtained for the study (10 shoulders). The sample size was determined with a security margin based on previous studies having demonstrated, with 6 shoulders, a sufficient power to generate statistically significant differences in translational and rotational stability.^12,15,23,25^ The absence of degenerative joint disease or previous ligamentous injury was confirmed by direct inspection and radiographs before testing. All specimens were acquired from the Geneva Faculty of Medicine from the body donation program after approval by the Cantonal Commission for Research Ethics (No. 2020-00598). All procedures were performed in accordance with the ethical standards of the institutional research committee.

**Figure 1.**
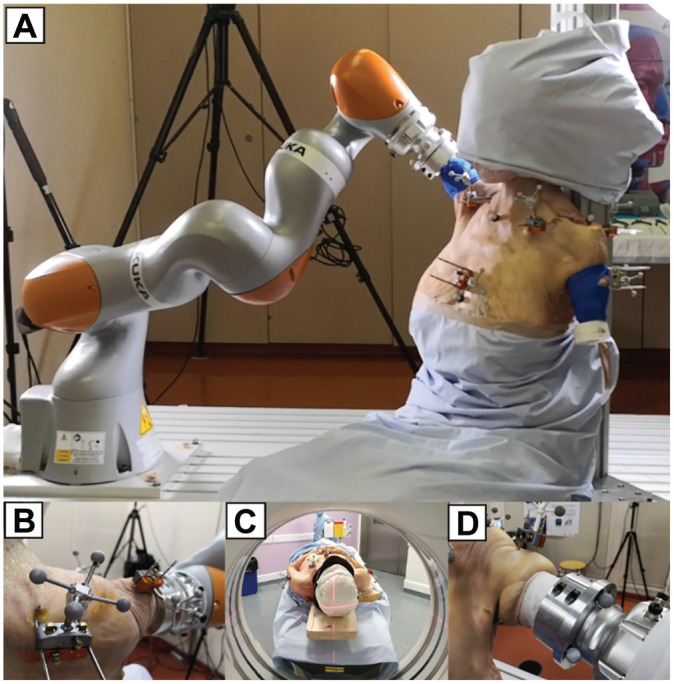
Testing setup. (A) Whole-cadaver setup with humerus mobilized by a robotic manipulator. (B) Clusters of reflective markers and related bone pins. (C) Computed tomography scanner. (D) Attachment of the humerus to the robotic manipulator.

Specimens were stored at −20° and thawed at room temperature overnight before preparation and testing. They were placed in a sitting position on a custom-made vertical support with wedges at cervical and lumbar levels to avoid any conflict between scapulas and support (Figure 1). Straps were tied at the cervical and lumbar levels to stabilize specimens. The specimen position and orientation were adjusted and rigidly secured to the table using clamps.

The 3-dimensional (3D) motions of the shoulder girdle bony segments were recorded by clusters of markers linked to intracortical pins. These pins were drilled through each segment following the protocol of Dal Maso et al.^11^ Stainless steel self-drilling pins (Hoffman 3; Stryker) were inserted in the thorax (diameter, 4 mm) and in the clavicle, scapula, and humerus (diameter, 3 mm). Pins were inserted as pairs and stabilized with clamps (Hoffman 3) to prevent axial rotation. Clusters of 4 reflective markers were secured to each pair of these pins (Figure 1).

### Testing Setup and Procedure

A set of humerus-standardized motions were applied by an industrial robot (KUKA LBR IIWA 14 R820; KUKA Robotics Corp) using the embedded KUKA Sunrise.OS (Version 1.10.0.8). To attach the limb, the humerus was transected, potted in a custom 3D-printed cylinder (acrylonitrile butadiene styrene thermoplastic polymer) with bone cement (Palacos LV; Heraeus), and rigidly mounted to the manipulator end effector (Figure 1). All soft tissues (eg, muscles, ligaments, and joint capsules) of the whole-shoulder girdle remained intact.

Humerus-standardized motions comprised flexion, abduction, internal-external rotation (at 0° of abduction), horizontal abduction, vertical traction, and horizontal compression. Motions were expressed as series of manipulator end-effector positions and orientations using custom-made software based on operator-induced humerus trajectories in the specimen-specific ranges of motion. The manipulator joint positions were then computed through an inverse kinematic procedure using ROS (Robot Operating System; Version 16.04.6 “Kinetic”) and the OpenRave IKFast solver (Version 0.9.0; http://openrave.org/docs/0.8.2/openravepy/ikfast/). Three cycles were recorded for each standardized motion using an 11-camera optoelectronic system sampled at 100 Hz (Oqus5; Qualisys), gap-filled, and filtered (second-order Butterworth filter; cutoff, 6 Hz). The same motion order was applied on each shoulder to ease the experimental protocol during the different assessed joint conditions (described hereafter).

### Surgical Joint Reconstructions

After intact joint condition testing, an incision was made in line with the long axis of the clavicle, exposing the superior part of the AC joint capsule. Complete transection of the articular capsule was carried out by sharp dissection. The distal clavicular attachment of the trapezius and anterior deltoid muscles was then released from the clavicle. CC ligaments were finally released at their clavicular attachment.

Shoulders were divided into 2 groups related to the surgical step sequence. Right shoulders were used for the CC-first group. CC drilling was realized by insertion of a 2.4-mm guide pin followed by overdrilling with a 4.5-mm reamer. A double-button system (ZipTight Fixation System; Zimmer Biomet) was inserted using a dedicated pusher and tensioned with interposition of a 10-mm spacer before AC stabilization with the selected bracing construct design. Restoration of the anatomic AC joint alignment was checked by visual inspection before construct tightening. Left shoulders were used for the AC-first group, in which AC stabilization was performed before the CC fixation.

**Figure 2.**
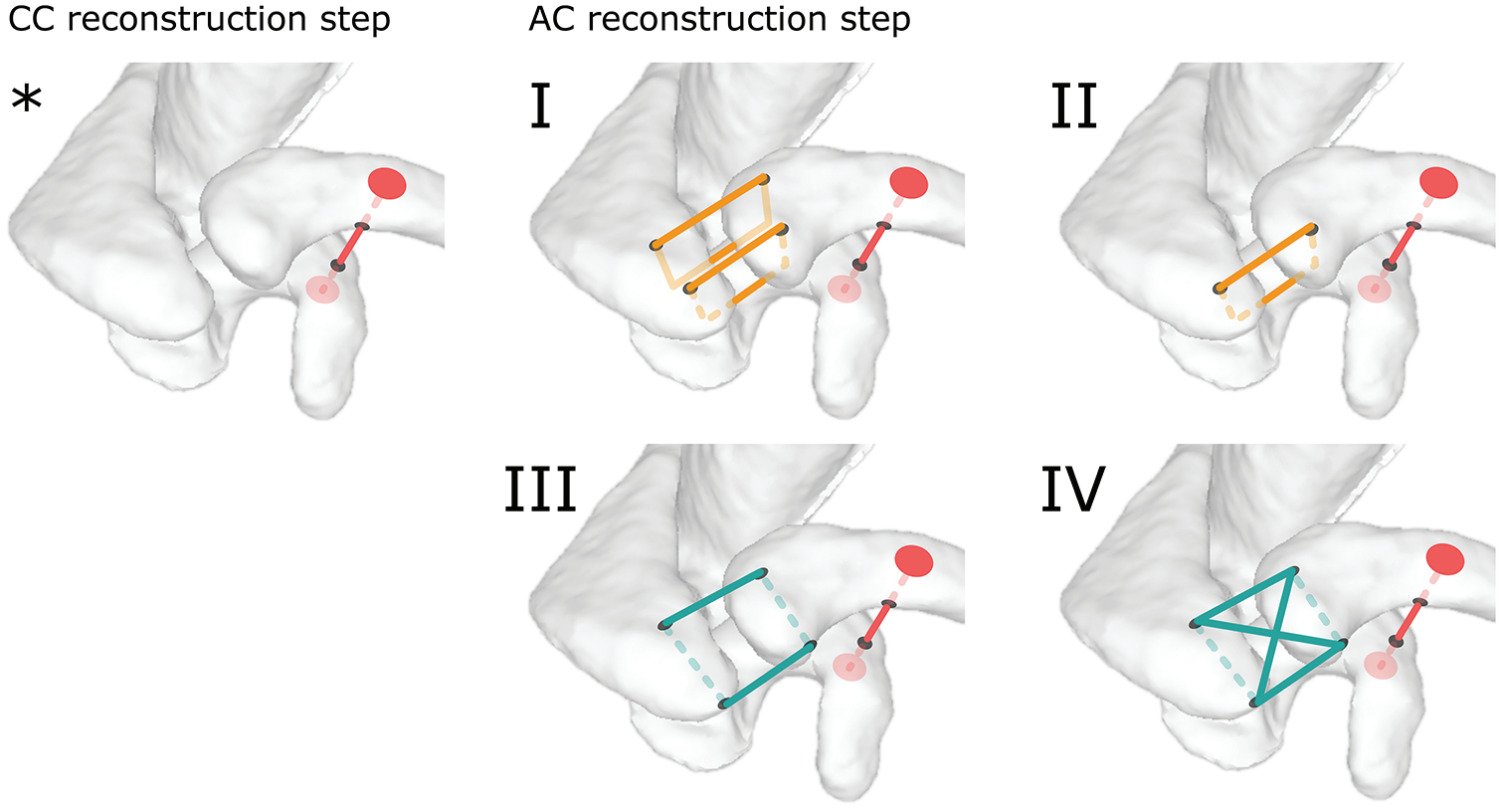
Surgical joint reconstructions. *Coracoclavicular (CC) only: CC drilling and double-button system installation only. (I) Bracing construct design 1: CC only + double vertical suture configuration. (II) Bracing construct design 2: CC only + single anterior vertical suture configuration. (III) Bracing construct design 3: CC only + single horizontal suture configuration. (IV) Bracing construct design 4: CC only + double horizontal suture configuration. AC, acromioclavicular.

In both the CC-first and AC-first groups, joint kinematics was assessed for the intact AC joint, an induced Rockwood type 5 lesion, isolated CC reconstruction, and 4 AC joint bracing construct designs (Figure 2). These designs were all based on suture loops and adapted from previously described techniques.

#### Bracing Construct Design 1: Double Vertical Suture

This bracing construct design is adapted from the vertical stabilization described by Cisneros et al.^9^ Two vertical 2.5-mm tunnels were drilled through the anterior acromion at the level of the anterior and posterior articular surface borders, and 10 mm lateral to the AC joint. Similarly, 2 vertical 2.5-mm tunnels were drilled through the distal clavicle 10 mm medial to the AC joint. Two suture tapes (BroadBand Tape; Zimmer Biomet) were passed through the posterior and anterior tunnels and tied in a Nice knot configuration,^5^ creating 2 vertical O-frame sutures (Figure 2, I).

#### Bracing Construct Design 2: Single Anterior Vertical Suture

This bracing construct design is a modification of design 1, keeping only the anterior vertical O-frame suture (Figure 2, II).

#### Bracing Construct Design 3: Single Horizontal Suture

This bracing construct design reproduces the horizontal O-frame suture configuration described by Hoffmeyer et al.^18^ Two horizontal (posterior to anterior) tunnels were drilled through the acromion 10 mm lateral to the AC joint and through the distal clavicle. A suture tape was passed through the posterior distal clavicle to the posterior acromion, and from the anterior acromion to the anterior distal clavicle, creating a horizontal O-frame suture (Figure 2, III).

#### Bracing Construct Design 4: Horizontal and Box Suture

This bracing construct design is a composite of design 3 and the X-frame suture configuration described by Hachem et al.^16^ A box suture tape was added to the horizontal O-frame suture. Additional suture tape was passed through the posterior distal clavicle to the anterior acromion and from the posterior acromion to the anterior distal clavicle to create a superior box suture (Figure 2, IV).

### Computed Tomography Scans

A CT scan was performed after specimen preparation and before the robotic manipulator procedure. Image acquisition extended from the sternoclavicular joint to the proximal third of the forearm, using a 16-row CT unit (CT LightSpeed VCT; GE HealthCare). The following scan parameters were used: field of view, 50 cm; matrix, 512 × 512; slice thickness, 0.6 mm; interval of reconstruction, 0.3 mm; 120 kVp, and 400 mA. Standard and bone filter reconstructions were acquired.

Segmentation of bones and reflective markers was achieved through a semiautomatic process using 3D Slicer (Version 4.10.2; https://www.slicer.org/).^13^ A surface model was then generated for each bone and related reflective markers using MeshMixer (Version 3.5.474; Autodesk). Bone coordinate systems were finally constructed by identifying through virtual palpation^39^ a set of anatomic landmarks according to the recommendations of the International Society of Biomechanics (ISB).^45^

### AC Joint Kinematics

**Figure 3.**
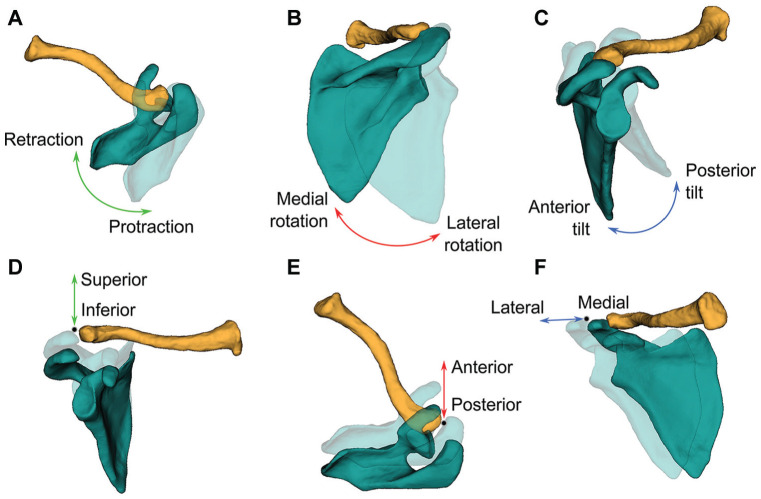
Acromioclavicular joint kinematics of the joint coordinate system.^45^ (A) Retraction-protraction of the scapula. (B) Lateral-medial rotation of the scapula. (C) Anterior-posterior tilt of the scapula. (D) Inferior-superior displacement. (E) Anterior-posterior displacement. (F) Lateral-medial displacement.

AC joint kinematics was described using Euler angles through a Y-X-Z order sequence according to the ISB recommendations.^45^ Displacements of the most dorsal point on the acromion of the AC joint were computed along the AC joint coordinate system axes (Figure 3). Thoracohumeral rotations, describing the motions of the humerus relative to the thorax, were defined by a plane of elevation, an elevation angle, and an axial rotation (Y-X-Y order).^45^ Both AC rotations and displacements were expressed as a function of the motion-related thoracohumeral angle. Data used to compute joint kinematics are available in a Zenodo repository (https://doi.org/10.5281/zenodo.8217257) and related code in a GitHub repository (https://github.com/fmoissenet/BLAB_Roboshoulder_toolbox).

### Data Reduction

Focusing on the effect of surgery on the AC joint alignment and stability, data reduction was conducted to compare the different joint conditions.

The AC joint alignment was expressed as the position of the most dorsal point on the acromion of the AC joint at minimal abduction between the intact and modified joint conditions. Inferior-superior, anterior-posterior, and lateral-medial displacements of this point were analyzed, as well as the 3D orientation of the scapula related to the clavicle (ie, retraction-protraction, lateral-medial rotation, and anterior-posterior tilt). For each outcome, the value measured for each experimental condition was subtracted from the value measured in the intact shoulder (defined as the reference). Differences were recorded between the intact and Rockwood 5 conditions as well as between the intact condition and bracing construct design in AC- and CC-first conditions. A value of zero thus indicates bone alignment identical to the intact shoulder. Data dispersion was assessed as interquartile range.

The AC joint stability was defined as the cumulative range of motion observed for each degree of freedom across all motions. Lower values of cumulative displacements and rotations correlate with an increased joint stability. Comparison with the intact shoulder was performed for descriptive purposes, as the surgical objective was to maximize the joint stability (here related to the minimization of joint motions) for the time of ligamentous healing.

### Statistical Analysis

The surgical step sequence effect on AC joint alignment was assessed across all shoulders and all bracing construct designs using a Mann-Whitney *U* test (unpaired samples).

The bracing construct design effect on AC joint stability was assessed across all shoulders and all surgical step sequences using a Friedman test (paired samples). The comparisons between Rockwood type 5 lesion condition and each bracing construct design condition were performed using a Fisher least significant difference procedure post hoc analysis.

## Results

**Figure 4.**
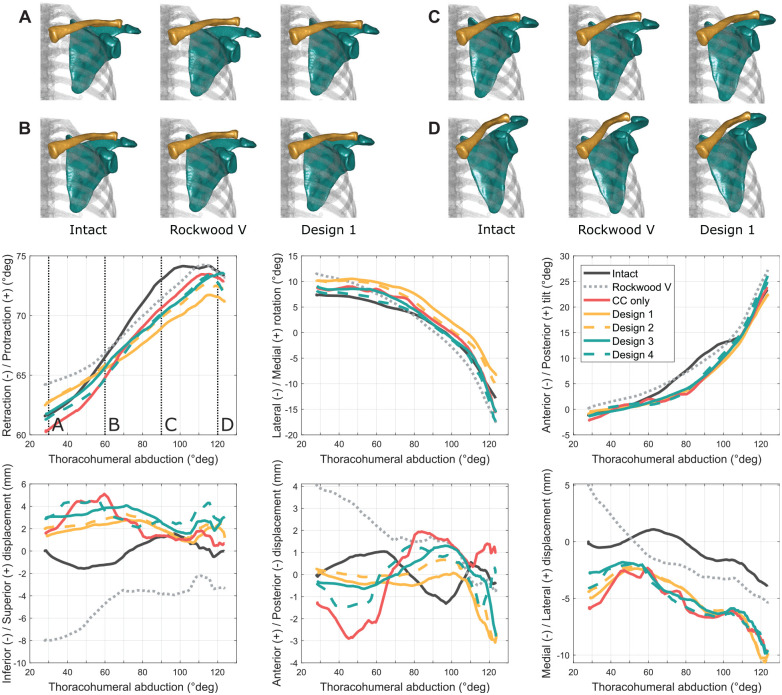
Acromioclavicular joint kinematics for each degree of freedom measured on 1 shoulder during thoracohumeral abduction. (A-D) Selected abduction levels corresponding to 30°, 60°, 90°, and 120° of abduction, respectively. CC only, joint only reconstructed with coracoclavicular double-button system; Intact, intact joint condition; Rockwood V, joint in Rockwood type 5 lesion condition.

The kinematic curve profiles obtained are illustrated in Figure 4 for each degree of freedom for 1 shoulder during abduction. The related scapula and clavicle bone pose (obtained by data fusion of bone segmentations and AC joint kinematics) were imaged for a set of discrete elevation angles and a set of joint conditions. The effects of the AC joint surgical step sequence and bracing construct design are reported and compared with the intact AC joint and Rockwood type 5 lesion conditions.

### Effect of Surgical Step Sequence

The difference between the intact joint and Rockwood type 5 lesion conditions on the AC joint alignment and orientation in the resting position is reported in Table 1. Friedman tests revealed a statistically significant difference for all degrees of freedom except retraction-protraction.

**Table 1:** Differences Between AC Joint Alignment and Joint Orientation (Scapula Related to the Clavicle) in the Resting Position Between Intact Joint and Rockwood Type 5 Lesion Conditions Across All Shoulders (n = 10)*^a^*

	Retraction - Protraction (+), deg	Lateral - Medial (+) Rotation, deg	Anterior - Posterior (+) Tilt, deg	Superior (+) - Inferior Displacement, mm	Anterior (+) - Posterior Displacement, mm	Lateral (+) - Medial Displacement, mm
Intact joint vs Rockwood type 5						
Intact joint median (95% CI)	59.1 (57.8 to 61.6)	2.3 (−2.2 to 7.4)	7.7 (2.9 to 9.7)	−0.0 (−0.0 to 0.0)	−0.0 (−0.0 to 0.0)	−0.0 (−0.0 to 0.0)
Rockwood type 5 median (95% CI)	62.4 (59.4 to 64.2)	4.1 (−3.3 to 10.4)	10.7 (5.5 to 13.8)	−8.7 (−13.0 to −6.4)	3.0 (1.9 to 4.2)	6.3 (3.5 to 8.0)
Median diff. (IQR diff.)	−3.3 (−1.1)	−1.8 (−4.1)	−3.0 (−1.5)	8.7 (−6.6)	−3.0 (−2.3)	−6.3 (−4.5)
*P* value*^b^*	.058	**.011**	**.002**	**.002**	**.011**	**.002**
AC-first vs CC-first						
AC-first median (95% CI)	1.9 (0.3 to 4.2)	−0.2 (−1.7 to 0.6)	10.1 (7.6 to 15.0)	2.2 (−0.8 to 4.1)	−0.8 (−2.9 to −0.1)	−1.0 (−5.5 to 1.0)
CC-first median (95% CI)	1.1 (0.0 to 2.0)	−2.1 (−3.4 to −0.2)	0.7 (−0.1 to 2.5)	3.7 (2.5 to 5.9)	−2.4 (−3.5 to −0.4)	−2.3 (−4.1 to −0.9)
Median diff. (IQR diff.)	−0.8 (−1.8)	−1.9 (0.9)	−9.4 (−4.8)	1.5 (−1.5)	−1.6 (0.3)	−1.3 (−3.4)
*P* value*^b^*	.172	.064	**.001**	**.041**	.365	.310

a AC, acromioclavicular; CC, coracoclavicular; diff., difference. The (+) indicates that the related motion is defined positive in the results, i.e. Retraction-Protraction (ȋ) means that positive values are related to protraction while negative values are related to retraction.

b Friedman test for paired samples. Bold *P* values indicate statistical significance (*P* ≤ .05).

**Figure 5.**
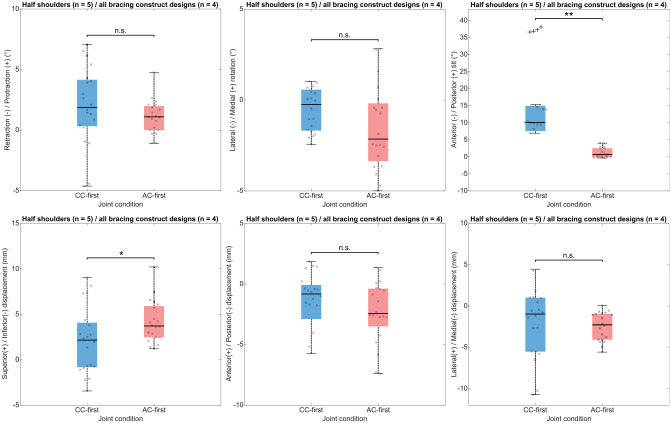
Box plots illustrating the surgical step sequence effect on acromioclavicular joint alignment. The colored box spans from the first quartile to the third quartile, with the black line representing the median. Whiskers extend from each quartile to the minimum and maximum values. Black crosses represent outliers. *P < .05; **P < .01. AC-first, inverted surgical step sequence; CC-first, coracoclavicular drilling and double-button system installation before acromioclavicular bracing construct design achievement; n.s., not significant.

The effect of the surgical step sequence (AC-first vs CC-first) on AC joint alignment and orientation in the resting position is reported in Figure 5 and Table 1. The Mann-Whitney *U* test revealed a statistically significant difference between the AC-first and CC-first sequences on joint reduction in anterior-posterior tilt (median difference, −9.4°; *P* = .001) and in inferior-superior displacement (median difference, 1.6 mm; *P* = .041). The AC-first sequence–related values demonstrated a lower dispersion than the CC-first sequence–related values for retraction-protraction (IQR difference, −1.8°), anterior-posterior tilt (IQR difference, −4.8°), inferior-superior displacement (IQR difference, −1.5 mm), and lateral-medial displacement (IQR difference, −3.4 mm).

### Effect of Bracing Construct Design

**Figure 6.**
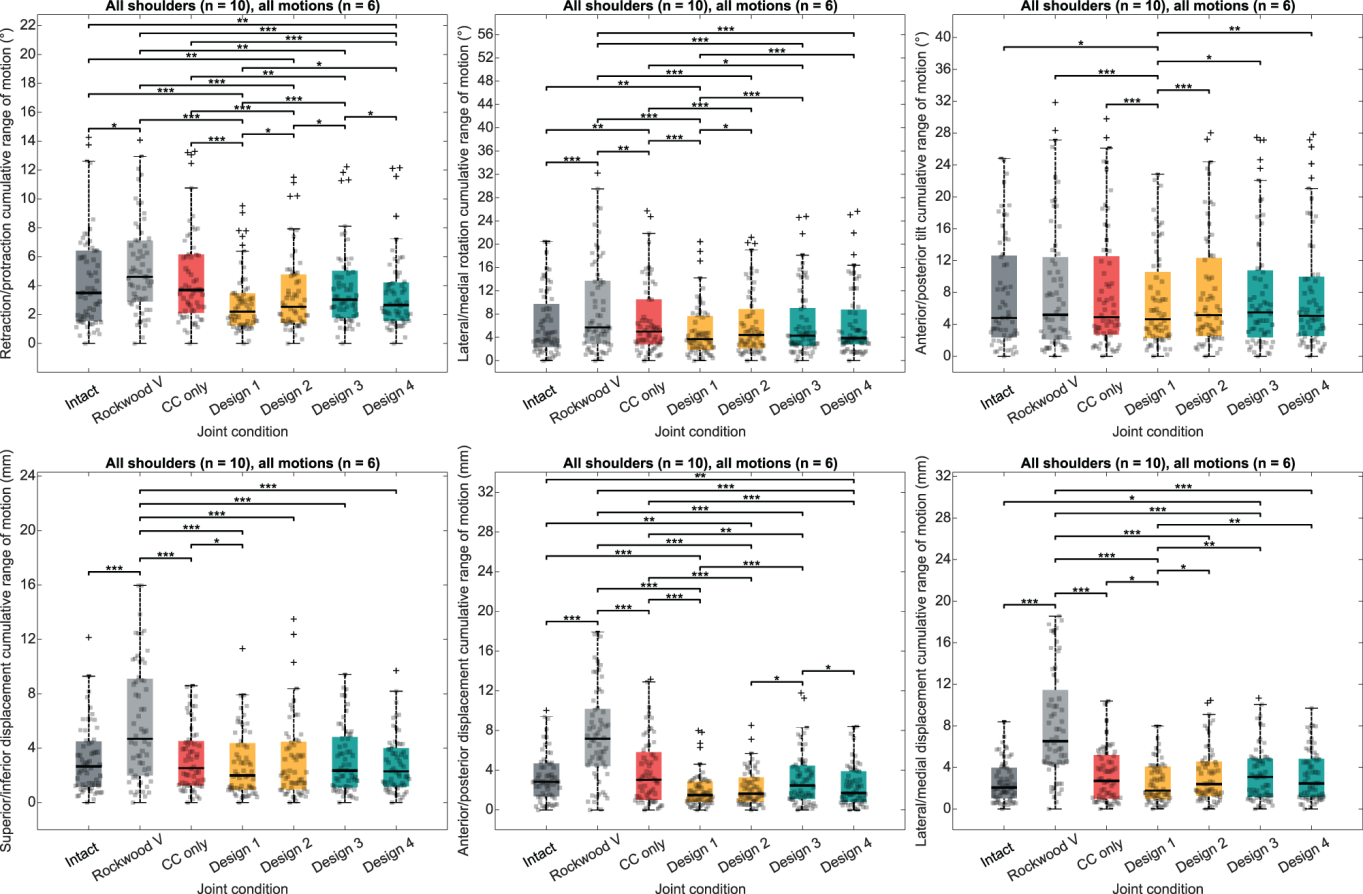
Box plots illustrating the bracing construct design effect on acromioclavicular joint stability. The colored box spans from the first quartile to the third quartile, with the black line representing the median. Whiskers extend from each quartile to the minimum and maximum values. Black crosses represent outliers. *P < .05; **P < .01; ***P < .001. CC only, joint only reconstructed with coracoclavicular double-button system; Native, intact joint condition; Rockwood V, joint in Rockwood type 5 lesion condition.

The effect of the AC joint bracing construct design on joint stabilization is demonstrated in Figure 6 and Supplementary Tables S1 to S3. The primary outcome was the cumulative range of motion observed for each degree of freedom across all motions (ie, flexion, abduction, internal-external rotation at 0° of abduction, horizontal abduction, vertical traction, and horizontal compression). The Friedman tests revealed a statistically significant difference in favor of bracing construct design 1 compared with the other designs in all rotations (ie, retraction-protraction, lateral-medial rotation, and anterior-posterior tilt), as well as along lateral-medial axis displacements.

## Discussion

The results of our pilot study confirmed our hypotheses of (1) an improved joint alignment when conducting AC reconstruction as the first surgical step and (2) an improved stability according to the bracing construct design, with the double vertical O-frame suture displaying the highest performances (bracing construct design 1).

### Kinematic Analysis of AC Lesions

The displacements between the clavicle and the acromion in the intact AC joint and Rockwood type 5 lesion equivalents have been reported previously in whole-shoulder models. Kurata et al^20^ recorded inferior-superior displacements of 9.5 ± 1.5 mm (range, 7.8 to 11.3 mm) and anterior-posterior displacements of −9.8 ± 3.0 mm (range, −13.8 to −6.5 mm) after a complete ligamentous sectioning. Peeters et al^31^ described a significant modification of the anterior-posterior tilt from 0° ± 8° to 2° ± 7° and inferior-superior displacements from −1 ± 3 mm to −11 ± 4 mm. The direction, amplitude of rotation, and displacements correlated with our findings.

### Effect of Surgical Step Sequence

Reduction of the AC joint before CC tunnel drilling has been proposed to optimize CC tunnel alignment.^38^ The improvement in radiological outcomes was later evidenced as a reduced loss of the CC distance ratio as well as a reduced tunnel widening.^37^ The reported incidence of postoperative loss of reduction greatly varies between authors, ranging from 0% to 80%.^6,10,34,36^ The reported risk factors for loss of reduction include a residual horizontal instability^38^ as well as a delayed surgical management, and an arthroscopic technique.^8^ To the best of our knowledge, our study is the first to report the effect of the surgical step sequence on the 3D joint alignment at rest for all 6 degrees of freedom. Conducting an AC reconstruction step before CC reconstruction confirmed the previous hypothesis of an improved AC joint alignment in anterior-posterior tilt. The significant differences between the surgical step sequences could be evidenced after only 3 repetitions of a complex motion sequence comprising flexion, abduction, internal-external rotation (at 0° of abduction), horizontal abduction, vertical traction, and horizontal compression. This finding supports the hypothesis that an improvement in joint alignment with the AC-first technique may ultimately allow for a reduction of postoperative complications, in terms of loss of reduction.

In our study, the AC joint reduction showed overcorrection in the superior-inferior, anterior-posterior, and lateral-medial directions compared with the intact AC joint after cyclic mobilization. The quality of reduction was determined by visual inspection of the superior acromial and clavicular articular border alignment, reflecting the clinical practice. Of note, the use of a 10-mm CC spacer was implemented to standardize the CC tensioning and avoid overreduction. Potential explanations for this overcorrection may be that the scapula was not repositioned to its normal state before surgical reconstruction, which may result in residual malalignment similar to that seen by altering the sequence of surgical steps.

Of note, this AC joint overreduction has recently been reported to improve the postoperative Rockwood classification^14,19^ without increasing 2-year postoperative complications.

### Effect of Bracing Construct Design

The whole-shoulder girdle model kinematic modifications in the presence of Rockwood type 5 lesions have been previously reported. Using the protocol of Oki et al,^28^ Pastor et al^30^ observed an anterior tilt of the clavicle of 1.1° (*P* = .012) and a lateral displacement of 2.71 mm (*P* = .017) in abduction. Direction, amplitude of rotation, and displacements correlated with our findings. Of note, all bracing construct designs increased the AC joint stability with respect to the intact condition, aligning with the defined surgical goals of (1) restoration of the anatomic joint alignment and (2) an internal fixation providing a temporary joint stabilization during capsuloligamentous healing before recovery of a full range of motion by the rehabilitation process.

Peeters et al^32^ reported a significant increase of protraction during abduction, from 16° ± 4° to 21° ± 5°, while implementing a rope and pulley system based on a protocol derived from Oki et al.^28^ The direction of displacement in this case was opposite the finding in our study as well as that of Pastor et al.^30^ A potential explanation could be heterogeneity in axis definition among authors.

Kinematic restoration after the AC joint reconstruction on a whole-shoulder girdle model has also been reported by Peeters et al^32^ when investigating the differences between AC, CC, and combined reconstruction techniques. They concluded that although each technique was able to restore elements of the joint kinematics, none completely restored the shoulder girdle to a preinjured state. Our study confirmed those findings, adding data on the optimization potential of the order of surgical steps and bracing construct design.

Interestingly, the effect of the AC capsule repair on joint stability and kinematics has been investigated using an isolated AC joint model. Dyrna et al^12^ reported the possibility of restoring the normal translational stability by adding an AC capsule augmentation, while a partial rotational instability remained. The tested individual constructs were found to display nonsignificant differences. The constructs of Dyrna et al^12^ partially overlapped the ones in this study. The analysis of the construct designs evidenced differences in the number and spatial distribution of bracing limbs spanning the joint. Dyrna et al^12^ retained a construct consisting of 1 to 2 spanning limbs mainly confined to one side of the joint. Our study investigated constructs comprising 2 to 4 spanning limbs, demonstrating an increased stability using the double vertical (4 limbs) construct. Unraveling kinematic differences by increasing the number and spatial distribution of spanning limbs might support the hypothesis that such constructs more closely reproduce the stabilizing effect of the intact AC capsule.

### Limitations

This study has several limitations. First, it was conducted on cadaveric specimens. The potential effect of age difference between specimens and patient population, as well as passive mobilization on results, has been already reported by Peeters et al.^31^ Second, the small sample size of cadaveric specimens did not allow for randomization of the testing sequences regarding the construct designs and motions. Third, biological healing cannot be included in the present protocol, and conclusions can only be drawn with respect to the primary stability of the constructs.^12^ Fourth, alternative techniques, not addressed in this study targeting simple suture bracing, consist of an anterior construct with suture tapes and screws as well as allograft/artificial graft reconstructions.^7,25,41^ We hypothesize that these techniques would exhibit biomechanical behavior similar to that of horizontal cerclage and simple vertical cerclage, respectively. Further collaborative studies are required for hypothesis verification. Fifth, the analysis was limited to the AC joint. Sixth, load to failure of clavicular and acromial tunnel drillings was not assessed. Although suture cutout and fractures were not observed, bone fragilization may result from this surgical procedure. Further studies on CC ligaments are required to better explore the effect of over- and underreductions on joint kinematics. Seventh, the joint stability assessment was simplified to a single outcome synthesizing the ranges of motion observed across all degrees of freedom. However, an evaluation for each degree of freedom remains possible using the present data set, which has been made publicly available through an open repository (https://doi.org/10.5281/zenodo.8146966).

## Conclusion

The implementation of surgical strategies comprising an AC joint reduction before CC tunnel drilling improved the final joint alignment. The choice of the AC surgical bracing construct design determines the amount of AC joint stabilization achieved in rotations and displacements, potentially affecting the final radiological and functional outcomes. The double vertical bracing technique achieved the highest construct stability over other tested designs during passive motion.

## Supplemental Material

sj-pdf-1-ajs-10.1177_03635465251349143 – Supplemental material for Acromioclavicular Fixation Before Coracoclavicular Tunnel Placement and Acromioclavicular Construct Design Improved Reduction and Stability in a Whole-Shoulder Girdle Model: A Pilot Study Supplemental material, sj-pdf-1-ajs-10.1177_03635465251349143 for Acromioclavicular Fixation Before Coracoclavicular Tunnel Placement and Acromioclavicular Construct Design Improved Reduction and Stability in a Whole-Shoulder Girdle Model: A Pilot Study by Nicolas Holzer, Pascal Boileau, Toby Baring, Jean-Yves Beaulieu, Noria Foukia, Michel Lauria, Stéphane Armand and Florent Moissenet in The American Journal of Sports Medicine

## Footnotes

# References 1, 12, 15, 17, 20, 24, 25, 30, 35
