# Supplementary material for: Acromioclavicular Fixation Before Coracoclavicular Tunnel Placement and Acromioclavicular Construct Design Improved Reduction and Stability in a Whole-Shoulder Girdle Model: A Pilot Study
Source: Am J Sports Med. 2025 Jun 26;53(9):2041–51. doi: 10.1177/03635465251349143 (PMC12235056; doi:10.1177/03635465251349143)
Supplement: sj-pdf-1-ajs-10.1177_03635465251349143 – Supplemental material for Acromioclavicular Fixation Before Coracoclavicular Tunnel Placement and Acromioclavicular Construct Design Improved Reduction and Stability in a Whole-Shoulder Girdle Model: A Pilot Study [file sj-pdf-1-ajs-10.1177_03635465251349143.pdf]

Acromioclavicular fixation before coracoclavicular tunnel placement and acromioclavicular construct design improved reduction and stability in a whole shoulder girdle model: a pilot study

Nicolas Holzer[1,2,3*], Pascal Boileau[4], Toby Baring[5], Jean-Yves Beaulieu[2,3], Noria Foukia[6], Michel Lauria[6], Stéphane Armand[7], Florent Moissenet[1,7]

[1] *Biomechanics Laboratory, Geneva University Hospitals and University of Geneva, Geneva, Switzerland.*
[2] *Department of Orthopaedics and Trauma Surgery - Geneva University Hospital, Geneva, Switzerland.*
[3] *Faculty of Medicine, University of Geneva, Geneva, Switzerland*
[4] *Institut de Chirurgie Réparatrice Locomoteur & Sport - Nice, France.*
[5] *Department of Orthopaedics, Homerton University Hospital, London, United Kingdom*
[6] *Geneva School of Engineering, Architecture and Landscape - HEPIA, Geneva, Switzerland.*
[7] *Kinesiology Laboratory, Geneva University Hospitals and University of Geneva, Geneva, Switzerland*

* Corresponding author:
Nicolas Holzer
Department of Surgery, Geneva University Hospitals, Geneva, Switzerland
Nicolas.Holzer@hug.ch

Supplementary material

Table S1 *– Cumulative ranges of motion (scapula related to the clavicle) observed for all degrees of freedom in intact joint, Rockwood type V injury, and all bracing construct designs across all shoulders (n = 10) and all motions (n = 6).*

	**Retraction-protraction**	**Lateral-medial rotation**	**Anterior-posterior tilt**	**Inferior-superior displacement**	**Anterior-posterior displacement**	**Lateral-medial displacement**
**Joint conditions**	**Median [CI95] (°)**	**Median (CI95) (°)**	**Median (CI95) (°)**	**Median [CI95] (mm)**	**Median (CI95) (mm)**	**Median (CI95) (mm)**
Intact	3.5 [1.6 to 6.4]	4.1 [2.3 to 9.7]	4.8 [2.4 to 12.6]	2.6 [1.2 to 4.5]	2.8 [1.4 to 4.7]	2.1 [1.0 to 4.0]
Rockwood V	4.6 [2.9 to 7.1]	5.7 [2.9 to 13.7]	5.2 [2.1 to 12.4]	4.7 [2.0 to 9.1]	7.1 [4.4 to 10.2]	6.5 [4.3 to 11.5]
CC only	3.7 [2.1 to 6.2]	4.9 [2.7 to 10.5]	4.9 [2.7 to 12.5]	2.5 [1.3 to 4.5]	3.0 [1.0 to 5.8]	2.7 [1.0 to 5.2]
Design 1	2.2 [1.3 to 3.5]	3.6 [1.9 to 7.7]	4.6 [2.3 to 10.6]	2.0 [1.0 to 4.4]	1.5 [0.8 to 2.9]	1.8 [1.0 to 4.1]
Design 2	2.5 [1.5 to 4.8]	4.3 [2.0 to 8.8]	5.2 [2.5 to 12.3]	2.2 [1.0 to 4.5]	1.6 [0.9 to 3.3]	2.4 [1.3 to 4.6]
Design 3	3.0 [1.8 to 5.0]	4.2 [2.6 to 9.0]	5.5 [2.3 to 10.8]	2.3 [1.1 to 4.8]	2.4 [1.1 to 4.5]	3.1 [1.2 to 4.8]
Design 4	2.6 [1.6 to 4.2]	3.8 [2.8 to 8.8]	5.1 [2.5 to 10.0]	2.3 [1.2 to 4.0]	1.7 [0.8 to 3.9]	2.5 [1.1 to 4.8]

CC: Coracoclavicular. CI95: Confidence Interval. Intact: Intact joint. Rockwood V: Rockwood V injury. CC only: CC drilling and double button system installation only. Design 1: CC only + double vertical suture configuration. Design 2: CC only + single anterior vertical suture configuration. Design 3: CC only + single horizontal suture configuration. Design 4: CC only + double horizontal suture configuration.

Table S2 – *Difference of cumulative ranges of motion (scapula related to the clavicle) observed for all rotational degrees of freedom between intact joint, Rockwood type V injury, and all bracing construct designs across all shoulders (n = 10) and all motions (n = 6).*

	**Retraction-protraction**		**Lateral-medial rotation**		**Anterior-posterior tilt**	
**Compared joint conditions**	**Median difference (IQR difference) (°)**	***p* value***	**Median difference (IQR difference) (°)**	***p* value***	**Median difference (IQR difference) (°)**	***p* value***
Intact - Rockwood V	*-1.1 (0.6)*	*p = 0.041*	*-1.7 (-3.4)*	*p < 0.001*	-0.4 (-0.1)	p = 0.394
Intact - CC only	-0.2 (0.8)	p = 0.061	*-0.8 (-0.3)*	*p = 0.003*	-0.1 (0.4)	p = 0.097
Intact - Design 1	*1.3 (2.7)*	*p < 0.001*	*0.5 (1.7)*	*p = 0.003*	*0.2 (1.9)*	*p = 0.013*
Intact - Design 2	*1.0 (1.6)*	*p = 0.001*	-0.2 (0.7)	p = 0.639	-0.4 (0.4)	p = 0.348
Intact - Design 3	0.5 (1.6)	p = 0.394	-0.1 (1.0)	p = 0.443	-0.7 (1.8)	p = 0.966
Intact - Design 4	*0.8 (2.2)*	*p = 0.004*	0.3 (1.5)	p = 0.217	-0.3 (2.8)	p = 0.639
Rockwood V - CC only	0.9 (0.2)	p = 0.865	*0.8 (3.0)*	*p = 0.006*	0.3 (0.5)	p = 0.418
Rockwood V - Design 1	*2.4 (2.0)*	*p < 0.001*	*2.1 (5.1)*	*p < 0.001*	*0.6 (2.1)*	*p = 0.001*
Rockwood V - Design 2	*2.1 (0.9)*	*p < 0.001*	*1.4 (4.0)*	*p < 0.001*	0.0 (0.6)	p = 0.932
Rockwood V - Design 3	*1.6 (1.0)*	*p = 0.004*	*1.5 (4.4)*	*p < 0.001*	-0.3 (2.0)	p = 0.418
Rockwood V - Design 4	*2.0 (1.6)*	*p < 0.001*	*1.9 (4.9)*	*p < 0.001*	0.1 (2.9)	p = 0.701
CC only - Design 1	*1.5 (1.8)*	*p < 0.001*	*1.3 (2.0)*	*p < 0.001*	*0.3 (1.5)*	*p < 0.001*
CC only - Design 2	*1.2 (0.7)*	*p < 0.001*	*0.6 (1.0)*	*p = 0.001*	-0.3 (0.1)	p = 0.469
CC only - Design 3	*0.7 (0.8)*	*p = 0.006*	*0.7 (1.4)*	*p = 0.030*	-0.6 (1.4)	p = 0.105
CC only - Design 4	*1.0 (1.4)*	*p < 0.001*	1.1 (1.9)	p = 0.088	-0.2 (2.4)	p = 0.233
Design 1 - Design 2	*-0.3 (-1.1)*	*p = 0.041*	*-0.7 (-1.0)*	*p = 0.012*	*-0.6 (-1.5)*	*p = 0.001*
Design 1 - Design 3	*-0.8 (-1.0)*	*p < 0.001*	*-0.6 (-0.7)*	*p < 0.001*	*-0.9 (-0.1)*	*p = 0.012*
Design 1 - Design 4	*-0.5 (-0.4)*	*p = 0.017*	*-0.2 (-0.2)*	*p < 0.001*	*-0.5 (0.9)*	*p = 0.003*
Design 2 - Design 3	*-0.5 (0.1)*	*p = 0.017*	0.1 (0.4)	p = 0.217	-0.3 (1.4)	p = 0.371
Design 2 - Design 4	-0.1 (0.7)	p = 0.733	0.5 (0.9)	p = 0.088	0.1 (2.3)	p = 0.639
Design 3 - Design 4	*0.4 (0.6)*	*p = 0.041*	0.4 (0.5)	p = 0.639	0.4 (1.0)	p = 0.670

* Fisher's least significant difference procedure post-hoc analysis (italic characters: $p \leq 0.05$).

CC: Coracoclavicular. Intact: Intact joint. IQR: interquartile difference. Rockwood V: Rockwood V injury. CC only: CC drilling and double button system installation only. Design 1: CC only + double vertical suture configuration. Design 2: CC only + single anterior vertical suture configuration. Design 3: CC only + single horizontal suture configuration. Design 4: CC only + double horizontal suture configuration.

Table S3 – *Difference of cumulative ranges of motion (scapula related to the clavicle) observed for all translational degrees of freedom between intact joint, Rockwood type V injury, and all bracing construct designs across all shoulders (n = 10) and all motions (n = 6).*

	**Inferior-superior displacement**		**Anterior-posterior displacement**		**Lateral-medial displacement**	
**Compared joint conditions**	**Median difference (IQR difference) (mm)**	***p* value***	**Median difference (IQR difference) (mm)**	***p* value***	**Median difference (IQR difference) (mm)**	***p* value***
Intact - Rockwood V	*-2.0 (-3.8)*	*p < 0.001*	*-4.3 (-2.5)*	*p < 0.001*	*-4.4 (-4.2)*	*p < 0.001*
Intact - CC only	0.1 (0.1)	p = 0.348	-0.2 (-1.5)	p = 0.147	-0.6 (-1.3)	p = 0.115
Intact - Design 1	0.6 (-0.1)	p = 0.201	*1.3 (1.2)*	*p < 0.001*	0.3 (-0.2)	p = 0.495
Intact - Design 2	0.4 (-0.2)	p = 0.609	*1.2 (0.9)*	*p = 0.001*	-0.3 (-0.4)	p = 0.081
Intact - Design 3	0.3 (-0.4)	p = 0.798	0.4 (-0.0)	p = 0.250	*-1.0 (-0.7)*	*p = 0.011*
Intact - Design 4	0.4 (0.5)	p = 0.798	*1.1 (0.2)*	*p = 0.001*	-0.4 (-0.7)	p = 0.055
Rockwood V - CC only	*2.1 (3.9)*	*p < 0.001*	*4.1 (1.0)*	*p < 0.001*	*3.8 (2.9)*	*p < 0.001*
Rockwood V - Design 1	*2.7 (3.7)*	*p < 0.001*	*5.6 (3.7)*	*p < 0.001*	*4.7 (4.0)*	*p < 0.001*
Rockwood V - Design 2	*2.5 (3.6)*	*p < 0.001*	*5.5 (3.3)*	*p < 0.001*	*4.1 (3.8)*	*p < 0.001*
Rockwood V - Design 3	*2.3 (3.4)*	*p < 0.001*	*4.7 (2.4)*	*p < 0.001*	*3.4 (3.4)*	*p < 0.001*
Rockwood V - Design 4	*2.4 (4.3)*	*p < 0.001*	*5.4 (2.7)*	*p < 0.001*	*4.0 (3.4)*	*p < 0.001*
CC only - Design 1	*0.5 (-0.1)*	*p = 0.027*	*1.5 (2.7)*	*p < 0.001*	*0.9 (1.1)*	*p = 0.024*
CC only - Design 2	0.3 (-0.2)	p = 0.147	*1.4 (2.4)*	*p < 0.001*	0.3 (0.9)	p = 0.865
CC only - Design 3	0.2 (-0.4)	p = 0.495	*0.6 (1.5)*	*p = 0.009*	-0.4 (0.5)	p = 0.327
CC only - Design 4	0.2 (0.4)	p = 0.495	*1.3 (1.7)*	*p < 0.001*	0.2 (0.5)	p = 0.733
Design 1 - Design 2	-0.2 (-0.1)	p = 0.443	-0.1 (-0.4)	p = 0.186	*-0.6 (-0.2)*	*p = 0.015*
Design 1 - Design 3	-0.3 (-0.3)	p = 0.125	*-0.9 (-1.3)*	*p = 0.001*	*-1.3 (-0.6)*	*p = 0.001*
Design 1 - Design 4	-0.3 (0.5)	p = 0.125	-0.2 (-1.0)	p = 0.217	*-0.7 (-0.6)*	*p = 0.009*
Design 2 - Design 3	-0.1 (-0.2)	p = 0.443	*-0.8 (-0.9)*	*p = 0.041*	-0.7 (-0.4)	p = 0.418
Design 2 - Design 4	-0.1 (0.7)	p = 0.443	-0.1 (-0.6)	p = 0.932	-0.1 (-0.4)	p = 0.865
Design 3 - Design 4	0.1 (0.9)	p = 1.000	*0.7 (0.3)*	*p = 0.033*	0.6 (-0.0)	p = 0.523

* Fisher's least significant difference procedure post-hoc analysis (italic characters: $p \leq 0.05$).

CC: Coracoclavicular. Intact: Intact joint. IQR: interquartile difference. Rockwood V: Rockwood V injury. CC only: CC drilling and double button system installation only. Design 1: CC only + double vertical suture configuration. Design 2: CC only + single anterior vertical suture configuration. Design 3: CC only + single horizontal suture configuration. Design 4: CC only + double horizontal suture configuration.
